# Is Vibration Training Good for Your Bones? An Overview of Systematic Reviews

**DOI:** 10.1155/2018/5178284

**Published:** 2018-11-04

**Authors:** Jorge Marin-Puyalto, Alba Gomez-Cabello, Alejandro Gonzalez-Agüero, Alejandro Gomez-Bruton, Angel Matute-Llorente, Jose A. Casajús, German Vicente-Rodríguez

**Affiliations:** ^1^Faculty of Health and Sport Science (FCSD), Department of Physiatry and Nursing. Universidad de Zaragoza, Ronda Misericordia 5, 22001 Huesca, Spain; ^2^GENUD (Growth, Exercise, Nutrition and Development) Research Group, Zaragoza, Spain; ^3^Instituto Agroalimentario de Aragón (IA2), Zaragoza, Spain; ^4^EXERNET Red de Investigación en Ejercicio Físico y Salud para Poblaciones Especiales, Spain; ^5^Centro Universitario de la Defensa, Zaragoza, Spain; ^6^Centro de Investigación Biomédica en Red de Fisiopatología de la Obesidad y Nutrición (CIBEROBN), Spain

## Abstract

Whole-body vibration (WBV) intervention studies and reviews have been increasing lately. However, the results regarding its effects on bone tissue in different populations are still inconclusive. The goal of this overview was to summarize systematic reviews assessing the effects of WBV training on bone parameters. Three electronic databases were scanned for systematic reviews and meta-analyses evaluating the effects of WBV on bone tissue. The search had no time restrictions and was limited to articles written in English. Vibration protocols and the main bone parameters included in each review were extracted. Methodological quality was assessed and analyses were conducted stratifying by age. 17 reviews and meta-analyses fulfilled the inclusion criteria. No increase or small improvements in bone mineral density (BMD) after WBV interventions were observed in reviews regarding postmenopausal women. One intervention study regarding young adults was included and reported no bone-related benefits from WBV. Most reviews including children and adolescents with compromised bone mass showed an improvement of BMD at lower limbs, lumbar spine, and whole body. In conclusion, WBV interventions seem to help children and adolescents with compromised bone mass to increase their BMD, but these improvements are limited in postmenopausal women and there is insufficient evidence for young adults. Further research is also needed to identify the ideal parameters of WBV training focused on bone health.

## 1. Introduction

Osteoporosis has been defined by the World Health Organization as a skeletal disease characterized by “low bone density and microarchitectural deterioration of bone tissue with a consequent increase in bone fragility and susceptibility to fracture” [1]. Osteoporosis and its related fractures are becoming an important public health concern, since they affect both quality of life and mortality of individuals [2] and generate health costs [3]. Even though this disease is more common among postmenopausal women, men can also suffer from it and, in some cases, its origin can be traced back to a low bone mass during adolescence and adulthood [4]. Therefore, the accrual of bone mineral density (BMD) is crucial in order to prevent or retard osteoporosis [5].

It has been widely tested that physical activity (PA) through lifetime and specific training programs have a beneficial influence on bone mass [6]. For this reason, the promotion of regular PA has been advocated as one of the main nonpharmacological measures for improving bone health [7].

Since Rubin et al. showed that low-magnitude high-frequency mechanical accelerations may produce a strong osteogenic response in animals [8] and humans [9], whole-body vibration (WBV) has become a topic of interest. Indeed, this marked increase in the use of WBV has led to the apparition of narrative [10–13], systematic [14–28], and also state-of-science [29] reviews focusing on different aspects, outcomes, or populations within this exercise training modality, as well as its safety for clinical practice.

WBV training uses high-frequency mechanical stimuli generated by a vibrating platform and transmitted through the body [30]. The platforms vary in the type of vibration produced (vertical or side-alternating) and the range of amplitudes and frequencies available [31]. The exact nature of the mechanism by which WBV training stimulates osteogenesis is still not certainly known [31].

The large number of studies published in the last years concerning the effects of WBV in different aspects and populations allows gathering the currently existing knowledge about the effects of this type of training on bone mass. However, there are still discrepancies among the reviews regarding the actual efficacy of this type of training for the improvement of bone mass throughout the diverse stages of life. Therefore, the main objective of this overview is to provide a global and summarized perspective of all current evidence regarding the effects of WBV training on bone mass.

## 2. Methods

### 2.1. Data Sources and Search Strategy

This study followed the overview methodology proposed by Smith et al. [32] and the framework provided by the Cochrane network [33].

Reviews were identified by searching electronic databases, scanning reference lists of reviews, and consultation with experts in the field. This search was applied to PubMed, SportDiscus, and the Cochrane Library. The search had no time restrictions set and was conducted up to and including 1 October 2018. The database-specific search terms were the following:

- PubMed: “vibration” [Title/Abstract] AND ((Meta-Analysis[ptyp] OR Review[ptyp]) AND “humans”[MeSH Terms])
- Cochrane Library: “vibration” in Title, Abstract, Keywords (Word variations have been searched) (only Cochrane Reviews and Other Reviews sections)
- SportDiscus: vibration AND review (only academic papers)

Bone-related search terms were not included in order to obtain a generic overview of all the reviews published concerning WBV and therefore have the certainty that no relevant articles were missing.

### 2.2. Review Selection

Two reviewers independently examined titles and abstracts. Relevant articles were obtained in full and assessed against the inclusion and exclusion criteria described below. Interreviewer disagreements were resolved by consensus. Arbitration by a third reviewer was used for unresolved disagreements.

### 2.3. Inclusion Criteria

1. Types of study: systematic reviews and meta-analyses concerning the effects of WBV training on bone mass. Within each systematic review, only the controlled trials measuring the outcomes later described were taken into consideration
2. Types of participants: children, adolescents, adults, and elderly populations (no age nor condition restrictions)
3. Types of outcome measured: bone mineral content (BMC) or BMD of whole body, lumbar spine, arm, hip (femoral neck, trochanter, intertrochanter, or Wards triangle subregions), bone architecture (from peripheral quantitative computed tomography (pQCT)), ultrasound parameters (Broadband Ultrasound Attenuation (BUA), Speed of Sound (SOS), and stiffness index), or metabolic biomarkers

### 2.4. Exclusion Criteria

1. Reviews in languages other than English
2. Nonsystematic reviews
3. Unpublished data
4. Reviews of studies with animals
5. Reviews focusing only on number of fractures, with no mention of variables obtained by imaging techniques
6. Meta-analyses that do not feature independent sets of effect sizes

### 2.5. Assessment of Methodological Quality

The evaluation of the methodological quality of the reviews was carried out using the AMSTAR tool [34], which has been validated as a mean to specifically assess the methodological quality of systematic reviews.

The overlapping of the included reviews was also considered by calculating the corrected covered area, a metric proposed by Pieper et al. [35], which measures the degree of overlap within a group of systematic reviews.

## 3. Results

### 3.1. Search Summary

1270 potentially relevant articles were retrieved. After reviewing the titles and abstracts, this total was reduced to 51. Of those reviews, 19 met the inclusion criteria and were included in a primary analysis.

**Figure 1:**
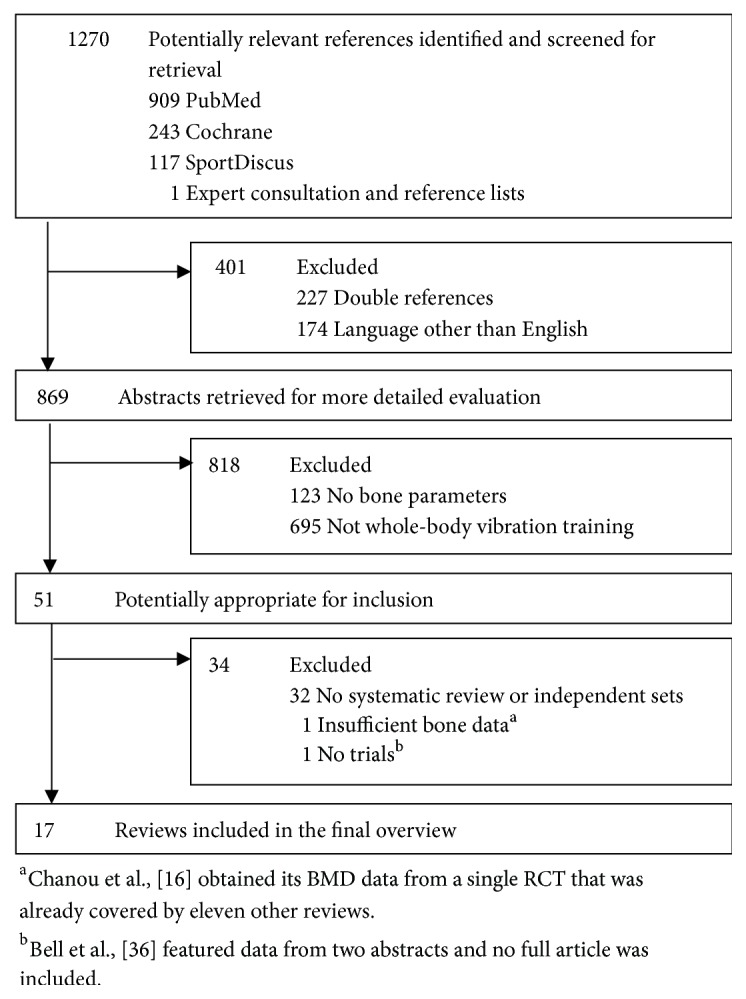
Flow chart diagram of the review selection.

Individual papers included in each review were listed. Studies related to bone variables were further examined and compared among reviews. This analysis allowed us to identify one review [16] that based its BMD results solely on one single trial (which was already covered by eleven of the other reviews) and another one [36] that included data solely from two abstracts. Therefore, the above-mentioned reviews were considered unlikely to add relevant data and were excluded from the final analysis (Figure 1).

### 3.2. Summary of Review Characteristics

The characteristics of the seventeen reviews included in the study are summarized in Table 1. In this table, the aim of the reviews and the search strategy followed by the authors are described. The number of studies included in these reviews, the total number of participants, the comparison interventions considered, and the duration range of these interventions are also reported. Finally, the main variables of interest for each review are listed.

### 3.3. Methodological Quality

Nine reviews [18, 21, 25–27, 37–40] fulfilled the requirements of seven or more of the eleven items evaluated, with one review [38] obtaining a perfect score and another one [27] achieving ten points. The other eight reviews [14, 20, 22, 23, 28, 29, 41, 42] obtained a total score of either five or six points. According to the classification proposed elsewhere [43, 44], five of the reviews [18, 27, 38–40] are considered of high quality and the rest of moderate quality. The complete results of the methodological quality assessment can be checked in detail in Table 2.

Regarding the overlapping among reviews, Table 3 shows the number of individual studies on the subject of bone mass which are repeated in each pair of reviews. The corrected covered area yielded a result of 16.8, which is considered as high. This outcome was expected, since most reviews cover the same target population.

### 3.4. Effects of WBV Training on Bone Mass in Different Populations

In order to facilitate the comparison between reviews, extracted data were sorted according to the studied population. Two reviews [21, 42] focused exclusively on children and adolescents with disabilities, eleven [14, 18, 22, 23, 26, 28, 37–41] on older populations, and four [20, 25, 27, 29] included children with disabilities, young adults, and elderly populations. Results from the latter reviews were subdivided into population categories, which included children and adolescents with disabilities, young adults, and older adults.

#### 3.4.1. Older Adults

The vast majority of the controlled trials that evaluated the effects of WBV on bone mass included in the present overview involved solely postmenopausal women, with no individual study focusing exclusively on older men. Therefore, the following results depict the effects of WBV on postmenopausal women.

*Hip and Femoral Neck. *All of the fifteen reviews that include older adults within their population of interest contain at least one primary study assessing the effects of WBV training on BMD at the hip or, more precisely, at the femoral neck.

All the studies included in the reviews state that WBV training is either positive or neutral regarding BMD at the hip or the femoral neck. Only one negative result from an individual study [45] has been found among all reviews. This article states that there is a slight loss of hip and femoral neck BMD after 8 months of WBV training. However, similar decreases were found in the exercise and control groups, with no group by time interaction, which results in WBV being neutral to BMD at this site.

Positive results predominate over neutral ones in nine of the fifteen reviews [14, 20, 22, 23, 25–29], whereas six reviews found more neutral than positive results [18, 37–41].

Six meta-analyses [18, 26, 37–40] have been conducted focusing specifically on the BMD at the hip and femoral neck. In one case [26], WBV training had an overall beneficial result when compared to sedentary controls. On the other hand, five meta-analyses [18, 37–40] found WBV to be neutral to hip BMD.

Three reviews [22, 25, 26] highlighted the positive role that WBV training plays in enhancing BMD at the hip and femoral neck, especially when compared to the results obtained for the lumbar spine. The rest of the researchers define the effects of WBV on BMD at the hip as small or nonsignificant, pointing out that there is a lack of consistency in the study designs within the literature, and state that WBV training could be useful as a complementary or alternative method to increase BMD at the hip for subjects who have difficulties following a standard training program.

*Lumbar Spine.* The lumbar spine is one of the main locations that have been studied due to its clinical relevance for the likelihood of fracture. All of the reviews include two or more individual studies that assessed BMD at this site.

No study has reported a detrimental effect of WBV training at the lumbar spine; however, neutral results clearly predominate over positive ones. Eight [18, 20, 23, 25–27, 39, 40] of the fifteen reviews have been unable to find positive results, while another seven found at least one study showing an enhancement of BMD at the lumbar spine. Nevertheless, all of them have found a greater number of studies reporting a lack of differences in lumbar spine BMD when comparing WBV training with other exercise modalities or controls.

All the authors that conducted a meta-analysis on the effect of WBV training on hip BMD performed the same analysis at the lumbar spine. Four of the meta-analyses [18, 26, 39, 40] showed no differences between WBV training and controls in lumbar spine BMD. Two of the latest meta-analyses included in this overview [37, 38] showed instead positive results of WBV training at this site, taking exclusively into consideration those studies with at least 6 months of WBV training.

*Other Sites.* The earliest assessment of cortical and trabecular volumetric BMD at the tibial midshaft was carried out by Russo et al. [46]. There have been conflicting interpretations among different authors, so this issue will be addressed in detail during the discussion of this overview.

A recent meta-analysis by Oliveira et al. [38] examined in depth the effects of WBV training on the volumetric BMD in postmenopausal women at both radius and tibia. No differences favouring any of the groups were found in either the primary analysis or the subsequent sensitivity subgroup analyses.

Whole-body BMD was assessed in two individual studies [30, 47] among the ones included within different reviews. Fjeldstad et al. [47] reported a detrimental effect on whole-body BMD for a combination of WBV training with resistance training, so it cannot be determined that these are solely due to the use of WBV. Verschueren et al. [30] showed no differences in whole-body BMD between the WBV and control groups after a 6-month period.

*Bone Turnover Markers.* Only five reviews [22, 23, 39–41] presented data regarding serum levels of bone turnover markers and extracted the information from six different original studies [30, 46, 48–51]. Four studies [30, 46, 48, 50] found no differences in the serum levels of these metabolic biomarkers among WBV and control groups. The study by Turner et al. [49] reported a reduction in the urinary levels of bone resorption markers (N-telopeptide X normalized to creatinine) following a 2-month WBV intervention. Finally, Corrie et al. found a greater increase in a bone formation marker (procollagen type 1 N-terminal propeptide) in subjects who underwent 12 weeks of WBV training compared to controls.

*Ultrasound Parameters.* Ultrasound parameters have only been considered in two reviews [38, 41], since the only study that evaluated the calcaneal region using this methodology was published two years ago by Slatkovska et al. [52]. In this study, a small but statistically significant decrease was found in the calcaneal broadband ultrasound attenuation following a 12-month intervention with low-intensity WBV, which implies a negative effect of WBV interventions.

*Optimal Parameters for WBV Training.* Several authors of reviews [14, 25, 27] suggest that more research is needed to establish the optimal parameters of vibration to improve or preserve BMD, but only three reviews [37, 38, 40] include subgroup meta-analyses to evaluate different vibration protocols.

In the meta-analysis by Ma et al. [37], the results for lumbar spine BMD favoured the WBV group when pooling the studies with low magnitude vibration (<1 g, as defined by the authors of the meta-analysis), but no differences were found in the studies with high-magnitude vibration (≥1 g). However, it is not clear that these effects are due solely to the magnitude of the vibration, given that the studies with low-magnitude vibration were as well those with a higher cumulative dose of vibration.

Oliveira et al. [38] carried out various subgroup meta-analysis, and they found significant improvements in lumbar spine BMD for the vibration group in the studies with side-alternating vibration, the studies where the subjects stayed with semiflexed knees during the WBV and the studies with either high frequency and low magnitude (>20 Hz, <1 g) or low frequency and high magnitude (≤20 Hz, ≥1 g).

In the review by Jepsen et al. [40], side-alternating platforms showed overall better results for lumbar spine BMD when compared to vertical vibration platforms.

#### 3.4.2. Young Adults

Two reviews [25, 27] included studies regarding the effects of WBV on BMD in young adults, and both of them retrieved the same original study [53]. In this randomized controlled trial, subjects that completed an eight-month intervention of WBV training did not show benefits over their control counterparts in BMC at any skeletal site measured (lumbar spine, femoral neck, trochanter, calcaneus, and distal radius). Volumetric BMD at the distal tibia and tibial shaft were also assessed, but no differences between groups were found after the WBV intervention.

Additionally, although the reviews did not analyze the results from bone remodelling markers, these parameters were effectively reported in the original study as not having changed following the intervention. This study did not include any ultrasound parameters [53].

In summary, the authors of reviews have not been able to find positive results linking WBV training with improved BMC or BMD in young, healthy adults from those reported in the only study found within this population [53].

#### 3.4.3. Children and Adolescents with Disabilities

A review conducted by Matute-Llorente et al. [21] explored the effects of WBV on bone mass in children and adolescents with disabilities. They claimed that even though the effect that this type of treatment exerts on body composition is not clear yet, it seems to provoke an improvement in bone health, since positive results predominate over neutral and negative ones within the studies they included, especially for lumbar spine BMD. The authors also acknowledge that the minimum dose of exposure to WBV required to elicit an optimal response is a topic that requires further research.

There is another review that explores the effects of WBV on children and adolescents with Down syndrome [42]. Different health parameters are analyzed within this review, but only one study regarding bone mineral status is included [54], which showed that WBV training has the potential to generate an increase in subtotal (whole body minus head) BMD above the regular growth in this population.

These are the only two reviews found which focused exclusively on children and adolescents with disabilities, but this population group is also taken into consideration in three other reviews.

On one hand, Rehn et al. [25] found increased volumetric BMD at the proximal tibia following WBV training [55, 56]. On the other, this improvement remained statistically nonsignificant at the lumbar spine [56]. Slatkovska et al. [27] obtained the same results for the proximal tibia. However, after performing a meta-analysis pooling both individual studies [55, 56], they found significant differences in the lumbar spine as well.

Finally, Wysocki et al. [29] consider that there is insufficient knowledge about the optimal target population for WBV training and therefore they do not reach different conclusions from the ones previously explained when focusing on postmenopausal women. They advise, thus, caution in making claims regarding this intervention, despite the fact that they report significant improvements in lumbar spine BMD when the adherence to WBV training is high in the only controlled trial focusing on children [55] which is included in their review.

All four reviews [21, 25, 27, 29] found positive results following a WBV intervention in BMC or BMD in various sites, including whole body and femoral neck and more consistently at the proximal tibia and lumbar spine. Serum levels of bone formation biomarkers were only included in one controlled trial and had a trend to increase after WBV training [57]. Ultrasound parameters have not been reported in any of the reviews.

## 4. Discussion

### 4.1. Summary of Main Findings

Concerning the effectiveness of WBV training for the improvement of BMD in postmenopausal women, the only significant results found are those in the hip or lower body with small effect sizes. The majority of studies that assessed BMD at the level of the lumbar spine found no changes. Several authors reported conflicting results, attributing differences to the variety of protocols used, with the longest training durations leading to changes in BMD [14, 25, 28, 37]. The necessity of long training periods in order to obtain results of minor clinic relevance yields WBV training as an ineffective method to improve bone mass. Nonetheless, it may be proven as a valid alternative for subjects unable to perform other types of training.

There is a lack of studies aiming to assess the evolution of BMD after WBV training in young adults, since only one publication [53] has been identified among the 31 papers evaluated by the fifteen reviews. The WBV program applied in that study did not affect BMD nor serum markers of bone turnover. However, more research is needed on this topic before issuing a recommendation for this population.

Improvements in BMD in children and adolescents with compromised bone mass have been found not only at the lower limbs but also at the lumbar spine and the whole body. Seven out of the nine studies included in a review that focused in this population [21] reported positive results at various sites. This is supported by the meta-analysis carried out by Slatkovska et al. [27], which found significant improvements in trabecular volumetric BMD at both the tibia and the spine following WBV training. The magnitude of the effect observed was higher when compared to postmenopausal women. This review [27] suggested that the growing skeleton of children and adolescents may be more sensitive to WBV training than other populations.

### 4.2. Discrepancies between Reviews

There were two papers regarding postmenopausal women which were interpreted differently across researchers. This can in part be the cause of discrepancies among reviews and inaccurate conclusions can be drawn when pooling the results if this is not taken into consideration. Therefore, the original documents of the individual studies were retrieved and further analyzed in order to clarify the actual results reported by the original studies.

According to most reviews [14, 18, 23, 27–29, 38], the study by Russo et al. [46] did not favour WBV training, since no changes in tibial vBMD were found in the vibration group after 6 months of intervention. However, the reviews by Merriman et al. and Rehn et al. [22, 25] point out that the control group suffered a decrease in the same parameter, and therefore that WBV training could be considered as a protective agent. The results from the original paper show indeed a significant decline in cortical BMD in controls, whereas it remained unchanged in the intervention group. Nonetheless, when contrasting the loss of BMD over time between the two groups, only a nonsignificant trend favoring vibration was found. Thus, the results from this paper do not support the claim by the latter reviews.

One of the most repeated studies within the different reviews is the one carried out by Verschueren et al. [30] which was included in a total of eleven reviews [14, 18, 20, 22, 25–29, 37, 39]. The reviews certify that this study presented an improvement in hip BMD following 6 months of WBV training, which is in concordance with the results from the original paper. However, when including this study in the context of a meta-analysis focused on the effects of vibration on hip or femoral neck BMD, different conclusions are drawn. In the meta-analysis performed by Lau et al. [18], this article is pooled with the one by von Stengel et al. [58] and in another meta-analysis by Sitjà-Rabert et al. [26] it is analyzed along with two other papers [59, 60]. In both cases, it is reported that the study by Verschueren et al. [30] shows that there are no results favouring vibration training over active exercise or controls.

### 4.3. Agreements and Disagreements with Other Studies or Reviews

Research is currently focusing on the different interventions that can help obtaining and maintaining an optimal bone health, and an overview examining the effects of exercise on bone status in female subjects has recently been published [61]. Even though it mentions WBV training, it only includes one systematic review [18], but it concludes that this type of training is not effective for protecting bone loss in postmenopausal women, which is in accordance with the results previously shown.

The findings presented in this overview are consistent with the results found by Gómez-Cabello et al. [62] in a study in which an eleven-week WBV training program was not able to improve BMD nor structure in the elderly. Moreover, a narrative review by Cheung and Giangregorio [63] showed that low-magnitude high-frequency WBV does not improve BMD and bone structure in postmenopausal women.

Similarly, Totosy de Zepetnek et al. [13] stated in their narrative review that the efficacy of WBV training among older adults is somewhat inconclusive, whereas this type of training has been shown to be anabolic to trabecular and cortical bone among young adults and children with low BMD or physical impairments. In a recent randomized controlled trial by Matute-Llorente et al. [54] which involved adolescents with Down syndrome, WBV training improved BMC and BMD of the whole body, lumbar spine, tibia, and radius.

These results seem to be in conflict with the ones presented by Rittweger [12] who suggested that WBV training could be more effective in the elderly than in young adults. However, this statement is supported by just two studies in postmenopausal women and one in young adults and therefore does not take into account several other studies that may find different outcomes.

There are three studies including different WBV protocols in order to compare vertical with side-alternating vibration [64], high-frequency with low-frequency vibration [65], and high-intensity with low-intensity WBV [66]. No differences between vibration protocols have been found in any of the studies. However, only one of them [65] compared frequencies, maintaining the rest of the variables stable. Therefore, further controlled trials focusing on one specific variable while controlling the rest of them are needed to clarify the optimal vibration parameters.

### 4.4. Strengths and Limitations

To the best of our knowledge, this is the first systematic overview of reviews regarding the effects of WBV training on BMC and BMD. This is a matter of increasing interest as it can be observed by the number of both narrative and systematic reviews related to this topic.

Most included reviews showed medium or high methodological quality and all of them were published in the last ten years. A high degree of overlapping studies within the reviews was identified and taken into account while evaluating the results, referring even to the primary study when discrepancies were found.

One of the main limitations of this overview is the difficulty encountered when trying to assess separately different vibration protocols, since only one review [28] assessed whether the vibration parameters were clearly established in the primary studies. Regarding this topic, the guidelines provided by Rauch et al. [67] could prove useful in the standardization of WBV intervention reports. In addition, different protocols are usually pooled together in the framework of the reviews. Moreover, some of the reviews included in this overview did not report the results from all the individual studies they included.

It was not possible to assess the publication bias as it was only considered by one of the reviews [27], even though it may affect this field of research, as suggested by Cardinale and Rittweger [31].

### 4.5. Implications for Future Research

Most authors agree that there is a necessity of finding the ideal vibration protocol to maximize the benefits of WBV. In order to achieve this goal, randomized controlled trials focused on identifying the specific role of vibration amplitude, frequency, and duration in the bone response to WBV are needed. Additionally, differences between subjects in the response to this training should be evaluated as well, since the optimal vibration parameters may vary within subjects [10].

As it has been mentioned, there is a scarcity of studies aimed at the evaluation of the effectiveness of WBV training in young adults. Future research might help understanding the evolution of the applicability of WBV to increase bone mineral content and density throughout life.

## 5. Conclusions

WBV training seems to be more effective in increasing BMC and BMD in children and adolescents with compromised bone mass than in postmenopausal women. Benefits of WBV training in BMC and BMD of postmenopausal women are limited to the lower limbs and are described as having little clinical relevance. Future research should establish the effects of this intervention in young adults as well as the precise vibration parameters required to elicit an optimal response in each population.

## Figures and Tables

**Table 1:** Summary of review characteristics.

Author, year	Aim	Search strategy	Number of WBV bone-related trials included (participants)	Comparison interventions	Duration of the interventions	Bone-related outcomes for which data were reported
**Older adults**						

Merriman, 2009	To assess the effectiveness of WBV on bone density, muscle performance, balance, and functional mobility	MEDLINE (1950-2007), CINAHL (1982-2007) No language restriction Intervention of at least 6 weeks	5 (251)	Control, placebo device, fitness exercise (<=40 min, 3/week), resistance exercise, physical therapy, walking	6-52 weeks	BMD (whole body, total hip, proximal femur, lumbar spine, cortical tibia) Bone turnover markers

Mikhael, 2010	To examine the effect of WBV on muscle or bone morphology and function	Medline, SPORTDiscus, AusportMed, CINAHL, AMED, WoS, Scopus English language Until 2009 (most databases)	3 (153)	Control, resistance, exercise, placebo device	6-52 weeks	BMC (lumbar spine) BMD (lumbar spine, tibia) Bone turnover markers

Lau, 2011	To determine whether WBV improves BMD and leg muscle strength	MEDLINE, CINAHL, EMBASE, PEDro, PubMed, Science Citation Index English language Exclusion criteria: Subjects diagnosed with a primary condition	6 (398)	Control, active exercise	6 weeks-18 months	BMD (total hip, lumbar spine)

Pollock, 2012	To assess studies which have investigated the outcome of repeated exposure to WBV	WoS, PubMed, EMBASE, CINAHL, PEDro, Cochrane Library English language Case reports and abstracts from conference presentations excluded	10 (719)	Control, placebo device, fitness exercise, walking, resistance training, wellness program	6-52 weeks	BMD (whole body, total hip, proximal femur, radius, lumbar spine)

Gómez-Cabello, 2012	To assess the effects of different training programmes in bone mass	MEDLINE & CENTRAL English and Spanish languages	10 (740)	Control, aerobic training, strength training and multicomponent training	6-18 months	BMC (whole body) BMD (lumbar spine, hip, femoral neck, distal radius, tibia)

Sitjá-Rabert, 2012	To analyze the efficacy and safety of WBV training	MEDLINE, EMBASE, CENTRAL, CINAHL, PeDro, PsychInfo English, French and Spanish languages	5 (247)	Control, exercise group (various modalities)	6-52 weeks	BMD (femoral neck, lumbar spine)

Ma, 2016	To evaluate the musculoskeletal effect of WBV	EMBASE, PubMed, Cochrane Central Register of Controlled Trials, ISI Web of Science, CNKI No date nor language restrictions Intervention of at least 6 months	8 (1014)	Control, resistance training, placebo device, walking	6-18 months	BMD (femoral neck, lumbar spine)

Oliveira, 2016	To analyze clinical trials that verified the effects of WBV on BMD in postmenopausal women	PubMed, Web of Science, LILACS, The Cochrane Library, PEDro No date or language restrictions Unpublished studies searched at clinicaltrials.gov	17 (1833)	Control, exercise group (various modalities)	6-18 months	BMD (lumbar spine, total hip, femoral neck, trochanter, Ward's area, tibia, radius)

Dionello, 2016	To review recent literature and highlight novel findings on the effect of WBV exercise on the BMD in women with postmenopausal osteoporosis without medications	PubMed, PEDro English language	11 (922)	Control, placebo device, wellness program, resistance training	2-18 months	BMD (whole body, lumbar spine, total hip, femoral neck) Bone turnover markers

Luo, 2016	To conduct a comprehensive quantitative analysis of WBV in patients with postmenopausal osteoporosis	MEDLINE, CINAHL, PEDro, CENTRAL	9 (625)	Control, wellness program, walking, resistance training	12-48 weeks	BMD (lumbar spine, femoral neck, total hip, Ward's triangle) Bone turnover markers

Jepsen, 2017	To address if WBV in adults over 50 years of age could affect the estimates of bone mass, architecture, and turnover biomarkers	PubMed, EMBASE, Cochrane English language	12 (1618)	Control, placebo device, exercise, wellness program	3-24 months	BMD (femoral neck, total hip, lumbar spine) Volumetric BMD (radius, tibia) Bone turnover markers

**Children and adolescents with disabilities**						

Matute, 2014	To summarize the current literature regarding the effects of WBV on health-related fitness parameters (children and adolescents with disabilities)	MEDLINE (PubMed), SPORTDiscus, EMBASE English language Published studies only	8 (338)	Control	8-52 weeks	BMC (whole body, lumbar spine) BMD (whole body, femoral neck, lumbar spine, tibia, femur) Stress endurance parameters Bone turnover markers

Saquetto, 2018	To verify the effects of WBV training on the muscle strength, bone mineral parameters, balance, and body composition of children and adolescents with Down's Syndrome	MEDLINE, LILACS, SciELO, PEDro, Cochrane No language restriction	1 (25)	Control	20 weeks	BMC (whole body, lumbar spine) BMD (whole body, lumbar spine) Volumetric BMD, cortical thickness (radius, tibia)

**No population restriction**						

Madou, 2008	To critique the research that has used WBV with special populations (elderly, postmenopausal women, and neurological patients)	ProQuest, IngentaConnect, Meditext, MEDLINE, Proquest5000, PubMed, SPORTDiscus, WoS, Health and Medical Complete, Google Scholar English, German, or Dutch languages (English abstract) Published in peer-reviewed journal	2 (99)	Control, resistance training	6-8 months	BMD (whole body, total hip, lumbar spine)

Rehn, 2008	To systematically review controlled studies that have explored the effects of WBV on BMD in humans	PubMed, Cinahl, Embase, Pedro, Amed Only controlled studies English language	8 (383)	Control, walking, placebo device, strength training	3-52 weeks	BMD (total hip, femoral neck, lumbar spine, tibia)

Slatkovska, 2010	To analyze the effects of WBV on BMD	MEDLINE, EMBASE, CINAHL, Cochrane, SPORTDiscus, ProQuest Dissertations, Theses Canada Portal No language restrictions Unpublished trials searched	8 (328)	Control, resistance training, placebo device, walking	6-52 weeks	BMD (total hip, lumbar spine)

Wysocki, 2011	To provide an overview of WBV therapy for the prevention and treatment of osteoporosis	MEDLINE, Cochrane Library, ACMD, CINAHL, CSA Physical Education Index, WoS, Physiotherapy Evidence Database, Academic Search Premier + gray literature 2000-2011 English language Exclusion criteria: population without low-BMD risk, subjects diagnosed with a primary condition	9 (617)	Control, walking, resistance training, wellness program, placebo device,	8-72 weeks	BMD (whole body, lumbar spine, total hip, femoral neck, trochanter, radius, tibia)

WBV: whole body vibration; BMC: bone mineral content; BMD: bone mineral density.

**Table 2:** Methodological quality of the included reviews according to the AMSTAR tool.

	“A priori” design	Duplicate study selection	Comprehensive search	Status of literature as inclusion criterion	List of studies (included and excluded)	Characteristics of studies	Scientific quality assessed	Scientific quality considered in conclusions	Methods to combine findings	Publication bias assessed	Conflict of interest	Total (out of 11)
Rehn, 2008	Y	Y	Y	Y	N	Y	Y	Y	NA	N	N	**7**
Madou, 2008	Y	CA	Y	Y	N	Y	Y	N	NA	N	N	**5**
Merriman, 2009	Y	CA	Y	N	N	Y	Y	Y	NA	N	N	**5**
Slatkovska, 2010	Y	Y	Y	Y	N	Y	Y	Y	Y	Y	Y	**10**
Mikhael, 2010	Y	CA	Y	Y	N	Y	Y	N	NA	N	Y	**6**
Lau, 2011	Y	Y	Y	Y	Y	Y	Y	Y	Y	N	N	**9**
Wysocki, 2011	Y	Y	Y	Y	N	Y	N	CA	NA	N	Y	**6**
Pollock, 2012	Y	CA	Y	Y	N	Y	Y	Y	NA	N	N	**6**
Gómez-Cabello, 2012	Y	Y	Y	Y	N	Y	N	CA	NA	N	Y	**6**
Sitjá-Rabert, 2012	Y	Y	Y	Y	N	Y	Y	N	Y	N	Y	**8**
Matute, 2014	Y	Y	Y	Y	N	Y	Y	Y	NA	N	Y	**8**
Ma, 2016	Y	Y	Y	Y	N	Y	Y	N	Y	N	Y	**8**
Oliveira, 2016	Y	Y	Y	Y	Y	Y	Y	Y	Y	Y	Y	**11**
Dionello, 2016	Y	Y	Y	N	N	Y	N	CA	NA	N	Y	**5**
Luo, 2016	Y	Y	Y	Y	N	Y	Y	Y	Y	N	Y	**9**
Jepsen, 2017	Y	Y	Y	Y	N	Y	Y	Y	Y	N	Y	**9**
Saquetto, 2018	Y	Y	Y	N	N	Y	Y	N	NA	N	Y	**6**

Y: yes; N: no; CA: cannot answer; NA: not applicable.

**Table 3:** Overlap within reviews.

Author, year	*1*	*2*	*3*	*4*	*5*	*6*	*7*	*8*	*9*	*10*	*11*	*12*	*13*	*14*	*15*	*16*	*17*
1. Madou et al., 2008	**2**	2	2	2	0	2	2	2	2	2	0	2	2	1	2	1	0
2. Rehn et al., 2008		**8**	5	8	3	5	6	5	5	5	2	4	5	1	3	2	0
3. Merriman et al., 2009			**5**	5	3	5	5	5	5	5	0	4	5	1	3	2	0
4. Slatkovska et al., 2010				**8**	3	5	6	5	5	5	2	4	5	1	3	2	0
5. Mikhael et al., 2010					**3**	3	3	3	3	3	0	2	3	0	1	1	0
6. Lau et al., 2011						**6**	6	6	6	5	0	5	6	2	4	3	0
7. Wysocki et al., 2011							**9**	8	7	5	1	5	6	3	4	3	0
8. Pollock et al., 2012								**10**	9	5	0	5	7	4	5	4	0
9. Gómez-Cabello et al., 2012									**10**	5	0	6	8	4	6	5	0
10. Sitjà-Rabert et al., 2012										**5**	0	4	5	1	3	2	0
11. Matute-Llorente et al., 2014											**8**	0	0	0	0	0	0
12. Ma et al., 2016												**8**	8	4	6	5	0
13. Oliveira et al., 2016													**17**	8	8	9	0
14. Dionello et al., 2016														**11**	7	6	0
15. Luo et al., 2016															**9**	6	0
16. Jepsen et al., 2017																**12**	0
17. Saquetto et al., 2018																	**1**

The values indicate the number of individual studies that are included in both reviews (row and column).

Bold characters show the number of RCTs for each review.

Corrected covered area: 16.8 (calculated as proposed by Pieper et al.).
